# Supplementary material for: Mapping the energy landscapes of supramolecular assembly by thermal hysteresis
Source: Nat Commun. 2018 Aug 8;9:3152. doi: 10.1038/s41467-018-05502-z (PMC6082911; doi:10.1038/s41467-018-05502-z)
Supplement: Supplementary file 1 — Supplementary Information [file 41467_2018_5502_MOESM1_ESM.pdf]

Supplementary Information

Mapping the energy landscapes of supramolecular assembly by thermal hysteresis

Robert W. Harkness V, et al.

Table of Contents

Supplementary Methods 3

Materials 3

Instrumentation 3

Acquisition of d(TG_4T) thermal hysteresis (TH) profiles 4

Acquisition of d(A_{15}) TH profiles 4

Temperature correction 5

Model-free analysis of TH datasets for generating 3D assembly maps 7

Global analysis of TG_4T TH profiles 10

Global analysis of TH profiles for CA-mediated poly(A) fiber formation 12

General interpretation of reaction orders for step-wise polymerization 16

Simulating TH profiles for classical nucleated supramolecular polymerizations 18

Calculating apparent reaction orders 19

Statistical analysis of errors 20

Supplementary Figures 23

Supplementary Tables 36

Supplementary References 37

Supplementary Methods

Materials

Cyanuric acid (CA), tris(hydroxymethyl)aminomethane (Tris), magnesium chloride hexahydrate ($MgCl_2 \cdot 6\ H_2O$), sodium cacodylate (NaCaco), sodium chloride (NaCl), glacial acetic acid and urea were used as purchased from Sigma-Aldrich. Boric acid was obtained from Fisher Scientific and used as supplied. Acrylamide/bis-acrylamide (40% 19:1) solution, ammonium persulfate and tetramethylethylenediamine (TEMED) were used as purchased from BioShop Canada Inc. Sephadex G-25 (super fine, DNA grade) was purchased from Glen Research.

Desalted $d(A_{15})$ and $d(TG_4T)$ oligonucleotides were purchased from Integrated DNA Technologies (IDT). $d(A_{15})$ was purified by denaturing polyacrylamide gel electrophoresis (PAGE) (8 mM urea, 1xTBE running buffer) and desalted with Sephadex G-25. $d(TG_4T)$ was used without further purification.

1xTBE (Tris-boric acid-EDTA) buffer was composed of 45 mM Tris, 45 mM boric acid and 2 mM EDTA at pH 8.3. 1xAcMg buffer was composed of 40 mM acetic acid, 7.6 mM $MgCl_2 \cdot 6\ H_2O$, with pH adjusted to 4.5. 1xNaCaco buffer was composed of 10 mM NaCaco and 100 mM NaCl (110 mM total Na^+) at pH 7.2. Buffers and samples were prepared with Milli-Q water.

Instrumentation

UV-Vis absorbance-based quantification of $d(A_{15})$ was performed on a Nanodrop Lite spectrophotometer from Thermo Scientific. Quantification of $d(TG_4T)$ was performed on an

Agilent Cary 300 UV-Vis spectrometer at 95 °C using a 10 mm path length quartz cuvette. DNA purification by PAGE was carried out on a 20 x 20 cm vertical acrylamide Hoefer 600 electrophoresis unit.

UV-Vis absorbance studies were performed using a 1 mm path length quartz cuvette on a Jasco-810 spectropolarimeter equipped with a Peltier temperature control unit and a water recirculator. Temperature verification on the instrument was performed with a handheld digital thermometer (Oakton) equipped with a fine gage thermocouple (Omega).

Acquisition of d(TG_4T) thermal hysteresis (TH) profiles

Samples contained 1 mM d(TG_4T) in 1xNaCaco buffer. The absorbance signals for annealing and melting were monitored at 295 nm over the 0 °C to 85 °C temperature range at different scan rates (0.2, 0.3, 0.5, 1, 1.5, and 2 °C min^{-1}). The rates were selected to ensure good separation between the curves. Samples were maintained at 85 °C for 10 minutes before annealing and at 0 °C for 10 minutes before melting. A layer of silicon oil was applied on top of the sample solution to minimize evaporation. A stream of nitrogen gas was supplied to the sample chamber to prevent condensation on the cuvette. Curves were obtained in triplicate.

Acquisition of d(A_{15}) TH profiles

Samples contained 50 μM d(A_{15}) and 15 mM CA in 1xAcMg pH 4.5. The absorbance signals for annealing and melting were monitored at 252 nm over the 2 °C to 65 °C temperature

range at different rates of temperature change (0.2, 0.5, 1, 2, 3, and 4 °C min^{-1}). The rates were selected to ensure good separation between the curves. Samples were maintained at 65 °C for 5 minutes before annealing and at 2 °C for 5 minutes before melting. A layer of silicon oil was applied on top of the sample solution to minimize evaporation. A stream of nitrogen gas was supplied to the sample chamber to prevent condensation on the cuvette with heating. Curves were obtained in triplicate.

Temperature correction

Thermal melting and annealing experiments can be subject to differences between the temperature of the solution in the experimental cuvette and the sample block temperature recorded by the instrument. Furthermore, this temperature difference changes as a function of experimental scan rate and additionally depends on the scanning direction (heating or cooling). Therefore, we measured the cuvette solution temperature with a digital thermocouple during heating and cooling scans as a function of scan rate, finding strongly linear correlations between the solution and block temperatures at all tested rates (Supplementary Figure 1). We corrected for the block temperature offset at each scan rate to a first approximation using

$$T_{\text{solution}} = mT_{\text{block}} + b \quad (1)$$

where m is the slope of the temperature correlation, found to be ~0.985 at all tested scan rates (Supplementary Figure 2a) and b is the temperature offset, i.e. the solution temperature when the block temperature is equal to 0 °C. We found that b varied linearly with the scan rate (dT/dt) (Supplementary Figure 2b), following the empirical relationship

$$b = -0.5578\frac{dT}{dt} + 1.9421 \quad (2)$$

TH profiles were subsequently corrected and resampled with linear interpolation (Supplementary Figure 2c,d) so that temperature points were identical for all scan rates (5-80 °C for TG_4T and 7-65 °C for poly(A) fibers in in 0.5 °C increments). The temperature corrected, resampled data were used for all analyses herein.

We additionally performed a validation of the temperature scan correction approach described above which can serve as a general test of temperature scan corrections obtained for thermal denaturation instruments. The procedure centers on measuring multi-scan rate thermal denaturation and renaturation datasets with a rapidly folding/unfolding sample, which in our case was an intramolecular G-quadruplex formed by the sequence 5'-AGGGTGGGIAGGGTGGGI-3'. For this type of system, the corrected folding and unfolding profiles should coincide (i.e. the TH should be approximately zero) at all tested scan rates. We measured heating and cooling profiles during which the instrument actively controlled the block temperature, while passively monitoring the sample temperature with a probe (Supplementary Figure 4a). Using this calibration, we performed the linear temperature correction described above (Supplementary Figure 4b). Importantly, this collapsed the profiles to their equilibrium positions, as expected for this type of rapidly folding system. The temperature correction was further validated by performing a separate experiment where the sample solution temperature was controlled directly via the integrated temperature probe (Supplementary Figure 4c). These profiles are nearly identical to the temperature-corrected data, demonstrating that our correction approach accurately reproduces the sample temperatures, without the need for an integrated temperature probe. Supplementary Figure 4a, illustrates that either a probe or temperature correction must be used in order to obtain reliable

data. Controlling only the block temperature without correcting for differences between block and sample temperatures leads to artifactually large amounts of TH (~8-10 °C), even for a rapidly folding sample where the system is in fact at thermal equilibrium throughout the experiment.

Model-free analysis of TH datasets for generating 3D assembly maps

Scan-rate dependent TH profiles were fit with linear baselines for the assembled (A_F) and monomeric, unfolded (A_U) signals according to

$$A_F(T) = m_F T + b_F \tag{3}$$

and

$$A_U(T) = m_U T + b_U \tag{4}$$

where m_U, m_F, b_U, and b_F are the unfolded and assembled baseline slopes and intercepts respectively. Using the baselines, the TH profiles were converted to fraction unfolded (θ_U)

$$\theta_U(T) = \frac{A(T) - A_F(T)}{A_U(T) - A_F(T)} \tag{5}$$

where $A(T)$ are the experimental thermal melting and annealing data. At low and high temperatures $\theta_U(T)$ takes limiting values of 0 and 1 respectively, corresponding to the completely assembled (= 0) or completely monomeric (= 1) states. We note that calculation of $\theta_U(T)$ relies on knowledge of both the assembled and disassembled baselines. This can present a challenge when large amounts of TH are present. For example, assembly of TG_4T reaches ~90% under our

experimental conditions with the remainder occurring during the low temperature equilibration period, meaning that low temperature baselines are not directly observed in the annealing scans. However melting scans provide well-defined assembled baselines, which we used as proxies for the assembled baselines of the annealing scans in order to calculate $\theta_U(T)$. The converse approach can be taken when the disassembled baselines cannot be directly observed in melting scans. For systems with extremely slow kinetics, the scan rate, monomer concentration, and solution conditions can be adjusted to promote assembly or disassembly in order to quantify $\theta_U(T)$.

The total concentration of nucleic acid in the experimental cuvette C_T is related to the concentrations of the monomeric and assembled states at each temperature by

$$C_T = [M](T) + N[F](T) \tag{6}$$

where N accounts for the number of monomers that reside within a folded assembly. The concentration of free monomers at each scan rate were calculated from the fraction unfolded assuming

$$[M](T) = \theta_U(T)C_T \,. \tag{7}$$

The slopes of the monomer concentration with respect to temperature $\frac{d}{dT}[M](T)$ were calculated numerically using rolling window regression where the derivative of a third-order polynomial fit to the calculated $[M](T)$ in a centered five point moving window is used with the experimental temperature increment of 0.5 °C to calculate the local slope (the movingslope function in MATLAB, https://www.mathworks.com/matlabcentral/fileexchange/16997-movingslope). The

rates of change of the monomer concentration were then obtained from the slopes and the scan rate:

$$\frac{d}{dt}[M](T) = \frac{dT}{dt}\frac{d}{dT}[M](T) . \tag{8}$$

The choice of polynomial order and window size was not found to dramatically influence the calculated values of $\frac{d}{dt}[M](T)$. Note that the scan rate is positive in the heating direction and negative in the cooling direction, leading to positive and negative $\frac{d}{dt}[M](T)$ in the heating and cooling directions respectively. The sets of scan rate dependent $\frac{d}{dt}[M](T)$ and $[M](T)$ from the annealing and melting portions of the experiment provide access to the temperature/reaction rate supramolecular assembly maps. These surfaces have larger reaction rates at faster temperature scan rates, as expected. The middle of the surface appears as a valley between the assembly and disassembly portions of the experiment and corresponds to concentrations close to their equilibrium values.

The surfaces are sliced with respect to temperature and a log-log analysis is performed according to Eqs. 1-4 in the main text. The intercepts correspond to effective rate constants for assembly and disassembly, however these contain contributions from a number of processes and are not meaningful for supramolecular pathway analysis. As guidelines for extraction of effective reaction orders using the model-free analysis presented here, we find the method requires (i) that there is adequate separation of the TH profiles for a given assembly or disassembly process as a function of temperature scan rate, e.g. for the assembly process, profiles collected at different scan

rates must differ from each other in the transition region by substantially more than the scatter due to experimental noise. (ii) The assembly and disassembly portions of TH data occur independently of each other. We suggest a roughly 3-fold difference in calculated annealing and melting rates in the middle of the annealing transition to ensure the observed orders reflect the pure assembly or disassembly processes. (iii) The concentration-rate plots are not performed using experimental data near or within the baseline regions. We recommend restricting the analysis to the ~10-90% fraction unfolded regions in order to obtain accurate orders.

Global analysis of TG_4T TH profiles

The TH profiles for TG_4T were globally fit assuming a model where quadruplex assembly proceeds via step-wise association of monomers[1] (Supplementary Figure 5c, Figure 2, Supplementary Figure 6c). The changes in concentration with respect to temperature are

$$\frac{d}{dT}[M]=\left(2k_{-1}[D]-2k_1[M]^2-k_2[M][D]+k_{-2}[Tr]-k_3[M][Tr]+k_{-3}[Q]\right)\frac{dt}{dT} \quad (9)$$

$$\frac{d}{dT}[D]=\left(k_1[M]^2-k_{-1}[D]+k_{-2}[Tr]-k_2[D][Tr]\right)\frac{dt}{dT} \quad (10)$$

$$\frac{d}{dT}[Tr]=\left(k_2[M][D]-k_{-2}[Tr]+k_{-3}[Q]-k_3[D][Tr]\right)\frac{dt}{dT} \quad (11)$$

$$\frac{d}{dT}[Q]=\left(k_3[Tr][M]-k_{-3}[Q]\right)\frac{dt}{dT} \quad (12)$$

where dt/dT is the inverse temperature scan rate. In what follows, the rate constants are assumed to be functions of temperature, and the concentrations of each species are assumed to be functions of temperature and scan rate, but we omit this notation for clarity. The temperature dependences of the rate constants are given by

$$k(T) = k_0 e^{\frac{E_a}{R}\left(\frac{1}{T_{ref}} - \frac{1}{T}\right)} \tag{13}$$

where k_0 is the rate constant at the reference temperature T_{ref} and E_a is the activation energy. In the global fit of the TG_4T TH profiles, the set of TG_4T assembly Eqs. 9-12 were numerically integrated using the ordinary differential equation (ODE) solvers in MATLAB (with ten minute pre-scan equilibrations) to obtain the concentrations of monomer, dimer, trimer, and tetramer as a function of temperature. The concentrations were converted to fraction unfolded and folded respectively using

$$C_T = [M] + 2[D] + 3[Tr] + 4[Q] \tag{14}$$

$$\theta_U = \frac{[M]}{C_T} \tag{15}$$

$$\theta_F = \frac{4[Q] + 3[Tr] + 2[D]}{C_T} \tag{16}$$

which permitted calculation of the thermal absorbance profiles as

$$A(T) = A_F(T)\theta_F(T) + A_U(T)\theta_U(T) \tag{17}$$

where $A_F(T)$ and $A_U(T)$ are the linear folded and unfolded absorbance baselines calculated according to Eqs. 3-4. The sets of TH profiles were fit by varying the kinetic parameters to minimize the RSS between the experimental and fitted absorbance data according to

$$RSS = \sum_{j=1}^{N} \sum_{k} \left(A_j^{\exp}(T_k) - A_j^{calc}(T_k, \xi) \right)^2 \quad (18)$$

Where $A_j^{\exp}(T_k)$ and $A_j^{calc}(T_k)$ are the jth experimental and fitted absorbance profiles respectively, T_k is the kth experimental temperature, $\xi = [k_1, k_{-1}, k_2, k_{-2}, k_3, k_{-3}, E_1, E_{-1}, E_2, E_{-2}, E_3, E_{-3}]$ are the rate constants at the reference temperature and activation energies governing assembly and disassembly of the tetramer.

We additionally fit models where monomers combine in a single step to yield tetramer[2] (Supplementary Figure 5a) or in a two-step dimer-of-dimers mechanism[3] (Supplementary Figure 5b). The single-step model gave poor fits (5.1-fold worse in terms of RSS) and failed to capture the assembly orders at all temperatures (Supplementary Figure 6a). The dimer-of-dimers model gave slightly worse fit (1.4-fold in terms of RSS) and effective order agreements (Supplementary Figure 6b). Therefore, the step-wise TG_4T assembly is our preferred model (Supplementary Figure 6c).

Global analysis of TH profiles for CA-mediated poly(A) fiber formation

The TH profiles for CA-mediated poly(A) fiber formation were globally fit with the Goldstein-Stryer model for cooperative self-assembly[4] (Supplementary Figure 7). The model assumes reversible, cooperative stepwise association of monomers (M) to form nuclei (M_s), which

then elongate to form fibers (M_N). The model has two distinct phases, where the pre-nucleus equilibria are governed by the nucleation rate constants k_{n+} and k_{n-}, and post-nucleus equilibria are governed by the elongation rate constants k_{e+} and k_{e-}. In order to limit the number of equations that must be numerically integrated, only fibers up to size N are explicitly described. Korevaar *et al.* showed that by treating all structures larger than the explicitly described size of N as a reversibly-formed fibril pool, increased numerical accuracy is obtained in solving this system of equations compared to straight truncation at a certain fiber length N[5, 6]. The Goldstein-Stryer model including the fibril pool for reversible self-assembly by Korevaar *et al.* is described by the following rate equations

Monomer

$$\frac{d}{dt}[M] = -k_{n+}[M]\left(2[M]+\sum_{i=2}^{s-1}[M_i]\right) - k_{e+}[M]\left(\sum_{i=s}^{N}[M_i]+[P]\right) + k_{n-}\left(2[M_2]+\sum_{i=3}^{s}[M_i]\right) + k_{e-}\left(\sum_{i=s+1}^{N}[M_i]+[P]\right) \tag{19}$$

Pre-nucleus oligomers

$$\frac{d}{dt}[M_i] = k_{n+}[M]\left([M_{i-1}]-[M_i]\right) + k_{n-}\left([M_{i+1}]-[M_i]\right) \tag{20}$$

Nucleus

$$\frac{d}{dt}[M_s] = k_{n+}[M][M_{s-1}] - k_{e+}[M][M_s] + k_{e-}[M_{s+1}] - k_{n-}[M_s] \tag{21}$$

Post-nucleus fibers

$$\frac{d}{dt}[M_i] = k_{e+}[M]\left([M_{i-1}] - [M_i]\right) + k_{e-}\left([M_{i+1}] - [M_i]\right) \tag{22}$$

Fiber length N

$$\frac{d}{dt}[M_N] = k_{e+}[M]\left([M_{N-1}] - [M_N]\right) + k_{e-}\left((1-\alpha)[P] - [M_N]\right) \tag{23}$$

Fibril number concentration

$$\frac{d}{dt}[P] = k_{e+}[M][M_N] - k_{e-}(1-\alpha)[P] \tag{24}$$

Fibril mass concentration

$$\frac{d}{dt}[Z] = k_{e+}[M]\left((N+1)[M_N] + [P]\right) - k_{e-}\left([P] + N(1-\alpha)[P]\right) \tag{25}$$

Where α is given by

$$\alpha = 1 - \left(\frac{[P]}{[Z] - N[P]}\right). \tag{26}$$

In our global fits, we assumed $k_{n+} = k_{e+}$[5]. Additionally, we allowed for non-zero nucleic acid folding ΔC_ps[7, 8] in the nucleation and elongation steps by including the $\Delta C_p^{\ddagger}$ parameter in calculating temperature-dependent activation energies

$$E_{n+}(T) = E_{n+}^{0} + \Delta C_p^{\ddagger}(T - T_{ref}) \tag{27}$$

where E_{n+}^{0} is the activation energy at the reference temperature. We have shown only the equation for the forward nucleation step for brevity. The set of differential equations for the Goldstein-Stryer model were numerically integrated as a function of temperature using the inverse scan rate dt/dT and the fractions of the unfolded monomer and polymerized states were calculated according to

$$\theta_U = \frac{[M]}{C_T} \tag{28}$$

$$\theta_F = \frac{\sum_{i=2}^{N} i[M_i] + [Z]}{C_T} \tag{29}$$

which permitted calculation of the thermal absorbance profiles as

$$A(T) = A_F(T)\theta_F(T) + A_U(T)\theta_U(T). \tag{30}$$

Global fits to the TH profiles for CA-mediated poly(A) fiber formation were carried out by minimizing the RSS in an identical manner to TG_4T. We varied the nucleus size to optimize the fit quality and agreement with the experimentally-determined effective assembly orders (Supplementary Figure 8). Out of the arrayed nucleus sizes, 2-4 gave excellent agreement with the data. While sizes of 2-4 are all physically realistic for poly(A) fiber formation, a nucleus of 3 fit the data best and therefore this is our preferred nucleus size. In addition, we varied the explicitly described fiber size N in order to verify that the fit results did not depend on its value. We found that annealing profiles simulated with a nucleus size of 3 and N = 50, 100, and 200 overlay, highlighting the utility of the approach developed by Korevaar *et al.*[5, 6] in global fitting of TH

datasets for supramolecular systems, as well as improving numerical accuracy and reducing computational time by allowing the use of smaller N values.

General interpretation of reaction orders for step-wise polymerization

The net flux of N-mer conversion to (N+1)-mers, Φ_N, depends on the concentrations of monomer, N-mer, and (N+1)-mer, (c_1, c_N, c_{N+1}, respectively), as well as the association rate of monomers and N-mers ($k_{on,N}$) and the dissociation rate of the (N+1)-mer ($k_{off,N+1}$) according to:

$$\Phi_N = k_{(\text{on,N})} c_1 c_N - k_{(\text{off,N+1})} c_{\text{N+1}} \tag{31}$$

for N>1. The flux of monomer to dimer conversion is given by:

$$\Phi_1 = 2\left(k_{(\text{on,1})} (c_1)^2 - k_{(\text{off,2})} c_2\right) \tag{32}$$

The total rate of monomer consumption is given by:

$$R = -\frac{\partial c_1}{\partial t} = \sum_{N=1}^{\infty} \Phi_{\text{N}} \tag{33}$$

and the effective order is

$$\frac{\partial ln\{R\}}{\partial ln\{c_1\}} = \frac{c_1}{R}\frac{\partial R}{\partial c_1} = \frac{c_1}{R}\sum_{N=1}^{\infty}\frac{\partial \Phi_{\text{N}}}{\partial c_1} = \frac{c_1}{R}\sum_{N=1}^{\infty}\frac{\Phi_N}{c_1}\frac{\partial ln\{\Phi_{\text{N}}\}}{\partial ln\{c_1\}}$$

$$= \sum_{N=1}^{\infty}\frac{\Phi_{\text{N}}}{R}\frac{\partial ln\{\Phi_{\text{N}}\}}{\partial ln\{c_1\}} \tag{34}$$

In other words, the effective order of monomer consumption is given by the weighted average of the orders of the individual fluxes ($\frac{\partial ln\{\Phi_{\mathrm{N}}\}}{\partial ln\{c_1\}}$) where the Nth weight ($\frac{\Phi_{\mathrm{N}}}{R}$) is the relative contribution of the Nth flux to the total rate. The order of each flux depends on how the populations of the *N*-mer and (*N*+1)-mer vary relative to the monomer concentration at a given temperature across the different scan rates, as well as the relative rate of depolymerization ($k_{\mathrm{off,N+1}}$).

$$\frac{\partial ln\{\Phi_{\mathrm{N}}\}}{\partial ln\{c_1\}} = \frac{c_1}{\Phi_{\mathrm{N}}}\frac{\partial \Phi_{\mathrm{N}}}{\partial c_1} = \frac{c_1}{\Phi_N}\left(k_{(\mathrm{on,N})}c_{\mathrm{N}} + k_{(\mathrm{on,N})}c_1\frac{\partial c_{\mathrm{N}}}{\partial c_1} - k_{(\mathrm{off,N+1})}\frac{\partial c_{\mathrm{N+1}}}{\partial c_1}\right)$$

$$= \frac{1}{\Phi_N}\left(k_{(\mathrm{on,N})}c_1 c_N + k_{(\mathrm{on,N})}c_1 c_{\mathrm{N}}\frac{\partial ln\{c_{\mathrm{N}}\}}{\partial ln\{c_1\}} - k_{(\mathrm{off,N+1})}c_{\mathrm{N+1}}\frac{\partial ln\{c_{\mathrm{N+1}}\}}{\partial ln\{c_1\}} + \left(k_{(\mathrm{off,N+1})}c_{\mathrm{N+1}}\frac{\partial ln\{c_{\mathrm{N}}\}}{\partial ln\{c_1\}} - k_{(\mathrm{off,N+1})}c_{\mathrm{N+1}}\frac{\partial ln\{c_{\mathrm{N}}\}}{\partial ln\{c_1\}}\right)\right)$$

$$= \frac{1}{\Phi_N}\left(k_{(\mathrm{on,N})}c_1 c_{\mathrm{N}} + \Phi_{\mathrm{N}}\frac{\partial ln\{c_{\mathrm{N}}\}}{\partial ln\{c_1\}} + k_{(\mathrm{off,N+1})}c_{\mathrm{N+1}}\left(\frac{\partial ln\{c_{\mathrm{N}}\}}{\partial ln\{c_1\}} - \frac{\partial ln\{c_{\mathrm{N+1}}\}}{\partial ln\{c_1\}}\right)\right)$$

$$= \frac{1}{\Phi_N}\left(\Phi_N + k_{(\mathrm{off,N+1})}c_{\mathrm{N+1}} + \Phi_{\mathrm{N}}\frac{\partial ln\{c_{\mathrm{N}}\}}{\partial ln\{c_1\}} + k_{(\mathrm{off,N+1})}c_{\mathrm{N+1}}\left(\frac{\partial ln\{c_{\mathrm{N}}\}}{\partial ln\{c_1\}} - \frac{\partial ln\{c_{\mathrm{N+1}}\}}{\partial ln\{c_1\}}\right)\right)$$

$$= 1 + \frac{\partial ln\{c_{\mathrm{N}}\}}{\partial ln\{c_1\}} + \frac{k_{(\mathrm{off,N+1})}c_{N+1}}{\Phi_{\mathrm{N}}}\left(1 + \frac{\partial ln\{c_{\mathrm{N}}\}}{\partial ln\{c_1\}} - \frac{\partial ln\{c_{\mathrm{N+1}}\}}{\partial ln\{c_1\}}\right) \quad (35)$$

Thus if depolymerization ($k_{(\mathrm{off,N+1})}c_{\mathrm{N+1}}$) is slow compared to the flux (Φ_{N}) and the concentration of the N-mer varies as the mth power of the monomer concentration across the scan rates , ($c_{\mathrm{N}} \propto (c_1)^m, \frac{\partial ln\{c_{\mathrm{N}}\}}{\partial ln\{c_1\}} = m$) , then the apparent order of the Nth flux ($\frac{\partial ln\{\Phi_{\mathrm{N}}\}}{\partial ln\{c_1\}}$) is *m*+1. With faster depolymerization rates, i.e. at values of [*M*] approaching the critical concentration, larger apparent reaction orders are obtained. The order of the monomer-to-dimer flux, Φ_1, is given by:

$$\frac{\partial ln\{\Phi_1\}}{\partial ln\{c_1\}} = \frac{c_1}{\Phi_1}\frac{\partial \Phi_1}{\partial c_1} = 2\frac{c_1}{\Phi_1}\left(2k_{(\mathrm{on},1)}c_1 - k_{(\mathrm{off},2)}\frac{\partial c_2}{\partial c_1}\right) = 2\frac{c_1}{\Phi_1}\left(2k_{(\mathrm{on},1)}c_1 - k_{(\mathrm{off},2)}\frac{c_2}{c_1}\frac{\partial ln\{c_2\}}{\partial ln\{c_1\}}\right)$$

$$= 2\frac{1}{\Phi_1}\left(2k_{(\mathrm{on},1)}(c_1)^2 - 2k_{(\mathrm{off},2)}c_2 + 2k_{(\mathrm{off},2)}c_2 - k_{(\mathrm{off},2)}c_2\frac{\partial ln\{c_2\}}{\partial ln\{c_1\}}\right)$$

$$= 2\frac{1}{\Phi_1}\left(\Phi_1 + k_{(\mathrm{off},2)}c_2\left(2 - \frac{\partial ln\{c_2\}}{\partial ln\{c_1\}}\right)\right)$$

$$= 2\left(1 + \frac{k_{(\mathrm{off},2)}c_2}{\Phi_1}\left(2 - \frac{\partial ln\{c_2\}}{\partial ln\{c_1\}}\right)\right) \qquad (36)$$

Simulating TH profiles for classical nucleated supramolecular polymerizations

Classical nucleated polymerizations were simulated according to the assumptions that (i) the monomer concentration changes only by addition to and subtraction from polymers longer than the nucleus, therefore the nucleus and pre-nuclear oligomers have small concentrations and are in rapid equilibrium with the monomer, (ii) polymer formation is irreversible, and (iii) the polymer elongation rate becomes zero when the monomer concentration reaches the critical concentration, $[M]_{critical}$. The equations for a classical nucleated polymerization[4] are

$$[s] = K_n^{\,s-1}[M]^s \qquad (37)$$

$$\frac{d}{dT}[M] = -k_{e+}[P]\left([M] - [M]_{critical}\right)\frac{dt}{dT} \qquad (38)$$

$$\frac{d}{dT}[P] = k_{e+}[s]([M]-[M]_{critical})\frac{dt}{dT} \quad (39)$$

Where $[s]$ is the concentration of the nucleus of size s, K_n is the equilibrium constant for nucleation $= k_{n+}/k_{n-}$, $[P]$ is the concentration of polymers larger than the nucleus, and the $[M]_{critical} = k_{e-}/k_{e+}$. Fraction unfolded TH profiles were simulated by numerically solving the concentration of monomer with the ODE solvers in MATLAB and dividing by the total monomer concentration C_T.

Calculating apparent reaction orders

We calculated theoretical reaction orders for TG_4T assembly for the step-wise association of monomers model approaching thermodynamic equilibrium with negligible concentrations of dimer and trimer ($[D]=(k_1/k_{-1})[M]^2$, $[Tr] = (k_2/k_{-2})[M][D]$). The rate of tetramer conversion to monomer is thus approximately equal to the rate of monomer conversion to tetramer. This is equal to the rate of monomer conversion to dimer ($k_1[M]^2$) multiplied by the net fraction of dimers (F_{DQ}) that continue forward to tetramer versus those that disassociate back to monomers

$$F_{DQ} = \frac{\left(\frac{k_2k_3[M]^2[D][Tr]}{k_{-2}[Tr]+k_3[M][Tr]}\right)}{k_{-1}[D]+\left(\frac{k_2k_3[M]^2[D][Tr]}{k_{-2}[Tr]+k_3[M][Tr]}\right)} \quad (40)$$

where the numerator of Equation 40 is the net rate of dimer to tetramer transition and $k_{-1}[D]$ is the net rate of the dimer to monomer transition[9]. The forward (monomer to tetramer) rate in the dynamic equilibrium is thus given by

$$R = k_1[M]^2 \left[\frac{\left(\frac{k_2 k_3 [M]^2 [D][Tr]}{k_{-2}[Tr] + k_3[M][Tr]} \right)}{k_{-1}[D] + \left(\frac{k_2 k_3 [M]^2 [D][Tr]}{k_{-2}[Tr] + k_3[M][Tr]} \right)} \right] = \frac{k_1 k_2 k_3 [M]^4}{k_{-1}k_{-2} + k_{-1}k_3[M] + k_2 k_3 [M]^2} \tag{41}$$

The order of R with respect to $[M]$ is thus

$$n_{app} = \frac{\partial ln\{R\}}{\partial ln\{[M]\}} = \frac{[M]}{R}\frac{\partial R}{\partial [M]}. \tag{42}$$

To assess the effects of changes in monomer concentration on the TH orders for TG_4T assembly, we simulated the apparent orders at fixed temperature and variable monomer concentration (Supplementary Figure 9). The change in R with respect to $[M]$ was calculated numerically using the movingslope function in MATLAB. The temperatures were held fixed at the lower and upper limits of 5 and 45 °C respectively (dark blue and dark red dashed lines in Supplementary Figure 9), while at each temperature, the monomer concentration was set at the average value used in the TH analysis at that temperature. Note that the monomer concentration was lower at low temperatures and higher at high temperatures. The theoretical orders at both limiting temperatures decrease with increasing $[M]$ (and T), as expected. This effect is overwhelmed in the experimental data by the shift in rate-determining barrier which leads to an increase in the apparent reaction order with increasing temperature.

Statistical analysis of errors

Errors in the global fit parameters were calculated using the variance-covariance matrix[10] given by

$$\hat{V} = \frac{RSS}{DF}(\hat{X}\hat{W}\hat{X}^T)^{-1} \quad (43)$$

where *RSS* is the residual sum of squared differences between experimental and fitted data points, *DF* is the degrees of freedom of the fit (*N* data points minus Φ parameters of the global fit) and $\hat{W}$ is a diagonal matrix of fitting weights, in this case all taken to be identically 1. $\hat{X}$ is a matrix of the first derivatives of the differences between the experimental and calculated data points ($A^{\exp}$ and A^{calc}), with respect to increments in each of the adjustable parameters (Φ_i). The element corresponding to the ith adjustable parameter and j^{th} data point is thus

$$X_{ij} = \frac{\partial\left(A_j^{\exp} - A_j^{calc}\right)}{\partial\Phi_i} \equiv \frac{\partial\alpha_j}{\partial\Phi_i} \quad (44)$$

where A_j^{calc} is evaluated at the optimized set of parameters, Φ. The elements were evaluated numerically according to

$$X_{ij} \approx \frac{A_j^{calc}(-\Delta) - A_j^{calc}(+\Delta)}{2\Delta} \quad (45)$$

where $A_j^{\text{calc}}(\pm\Delta)$ is the jth data point calculated with all adjustable parameters set to their optimized values except, for the ith parameter, which is incremented by $\pm\Delta$. For a global fit with *N* data points and *M* adjustable parameters this gives

$$\hat{X} = \begin{bmatrix} \frac{\partial\alpha_1}{\partial\Phi_1} & \cdots & \frac{\partial\alpha_N}{\partial\Phi_1} \\ \vdots & \ddots & \vdots \\ \frac{\partial\alpha_1}{\partial\Phi_M} & \cdots & \frac{\partial\alpha_N}{\partial\Phi_M} \end{bmatrix} \quad (46)$$

The diagonal elements in $\hat{V}$ are the variances of the optimized fit parameters, while the off-diagonal elements are the covariances between the errors of the optimized parameters. Errors in fitted parameters were taken as the square root of the variances.

Supplementary Figures

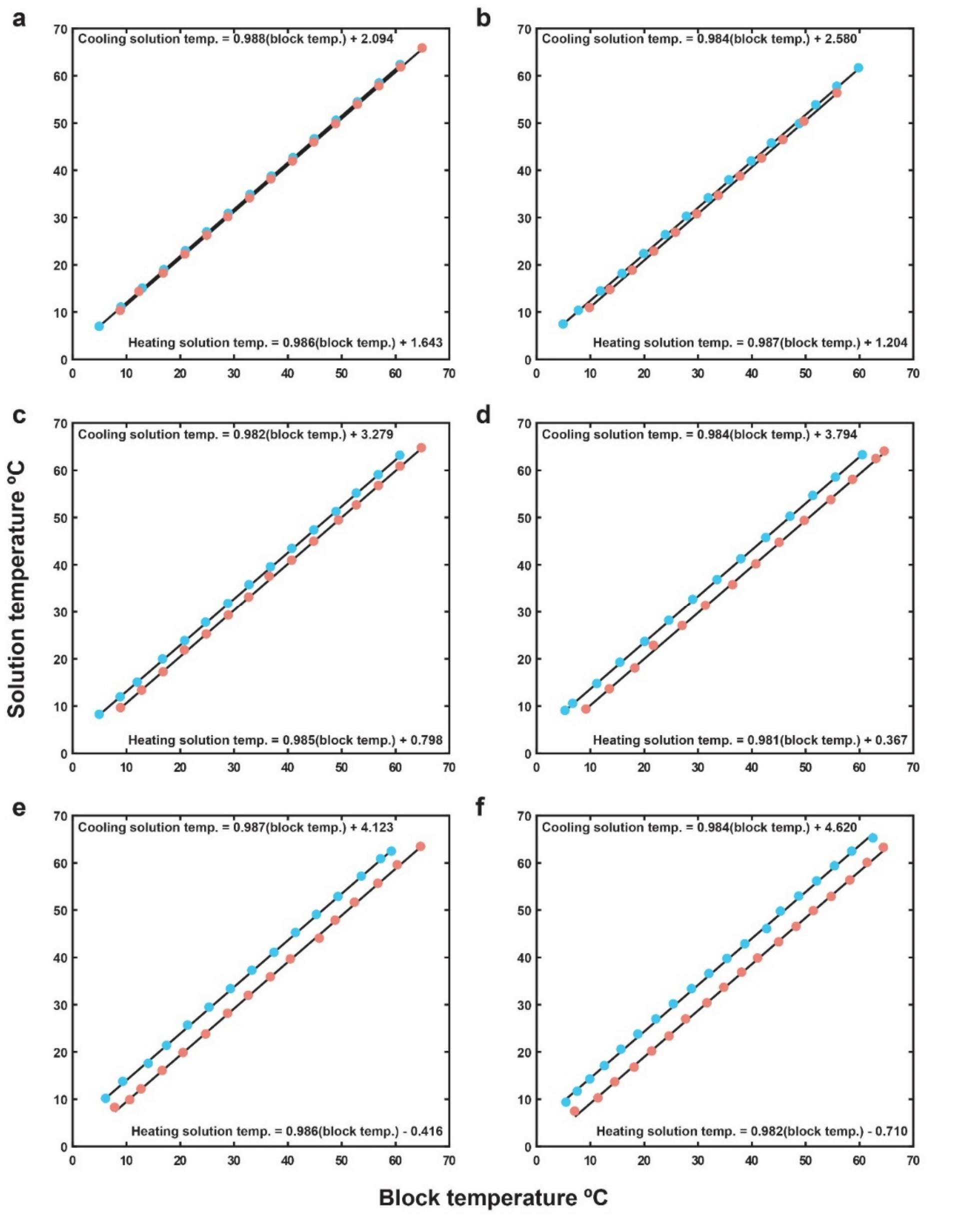

Supplementary Figure 1. Solution versus block temperature as a function of scan rate. (a) ±0.5 °C min^{-1} scan rates. (b) ±1 °C min^{-1} scan rates. (c) ±2 °C min^{-1} scan rates. (d) ±3 °C min^{-1} scan rates. (e) ±4 °C min^{-1} scan rates. (f) ±5 °C min^{-1} scan rates. In all panels, the cooling and heating scan temperatures are shown as blue and red circles respectively. Linear fits to the scan temperatures are shown as black lines. The parameters corresponding to the linear fits of the cooling and heating scan temperatures are shown in the top left and bottom right of each panel respectively.

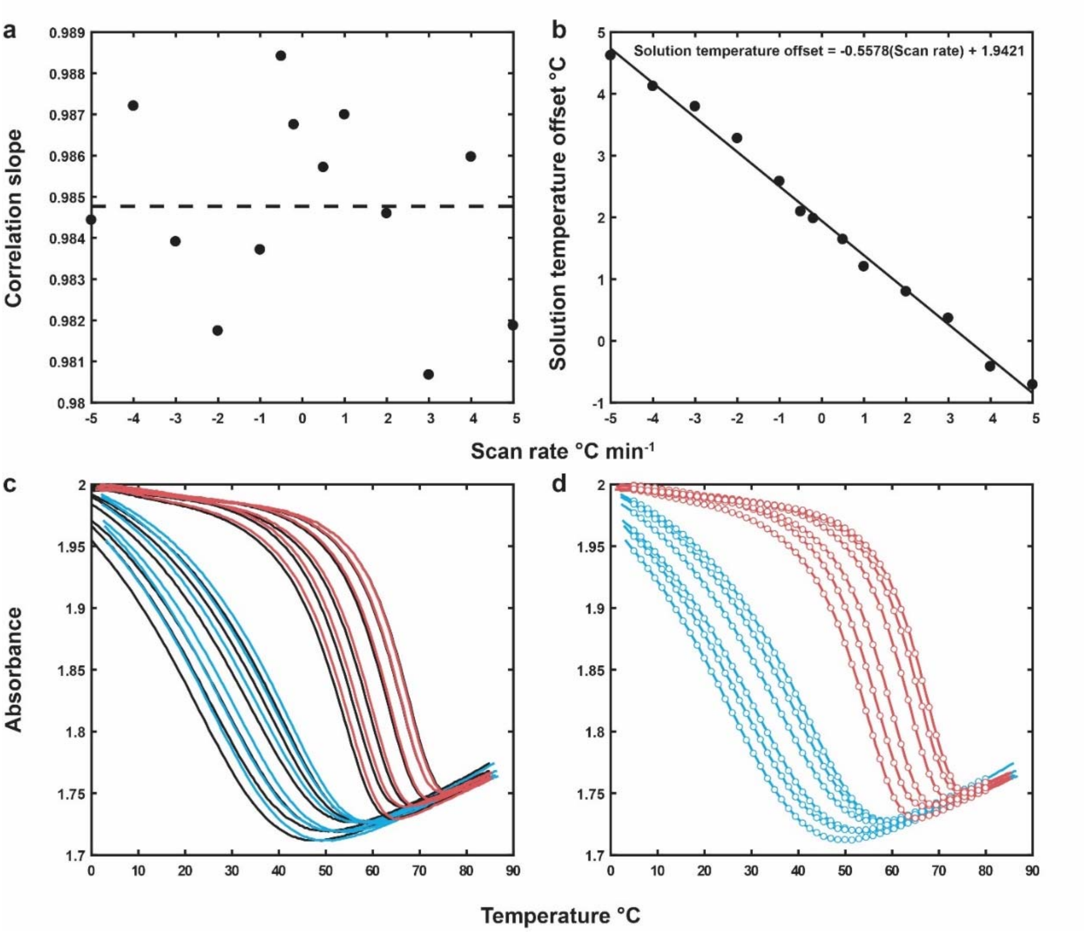

Supplementary Figure 2. Temperature correction of TH data. (a) Slopes from the correlations in Supplementary Figure 1 as a function of temperature scan rate. The dashed black line indicates the mean slope (~0.985). (b) Solution temperature offset as a function of scan rate. Offsets were taken as the intercepts from the linear fits to the correlations in Supplementary Figure 1. A linear fit to the solution temperature offsets as a function of scan rate is shown as a black line, with the corresponding fit parameters given in the top right of the panel. (c) Uncorrected (black lines) and temperature corrected TG_4T thermal hysteresis profiles (blue and red lines for annealing and melting respectively). The correction was performed using $T_{solution} = 0.985(T_{block})$ + offset where the offset was calculated from the equation given in Supplementary Figure 2b. (d) Linearly interpolated TH profiles (blue and red empty circles for annealing and melting respectively) overlaid with the temperature corrected profiles from Supplementary Figure 2c. Only every third interpolated point is shown for clarity. The interpolation was performed to place the corrected data on the same temperature domain, from 5-80 °C in 0.5 °C increments.

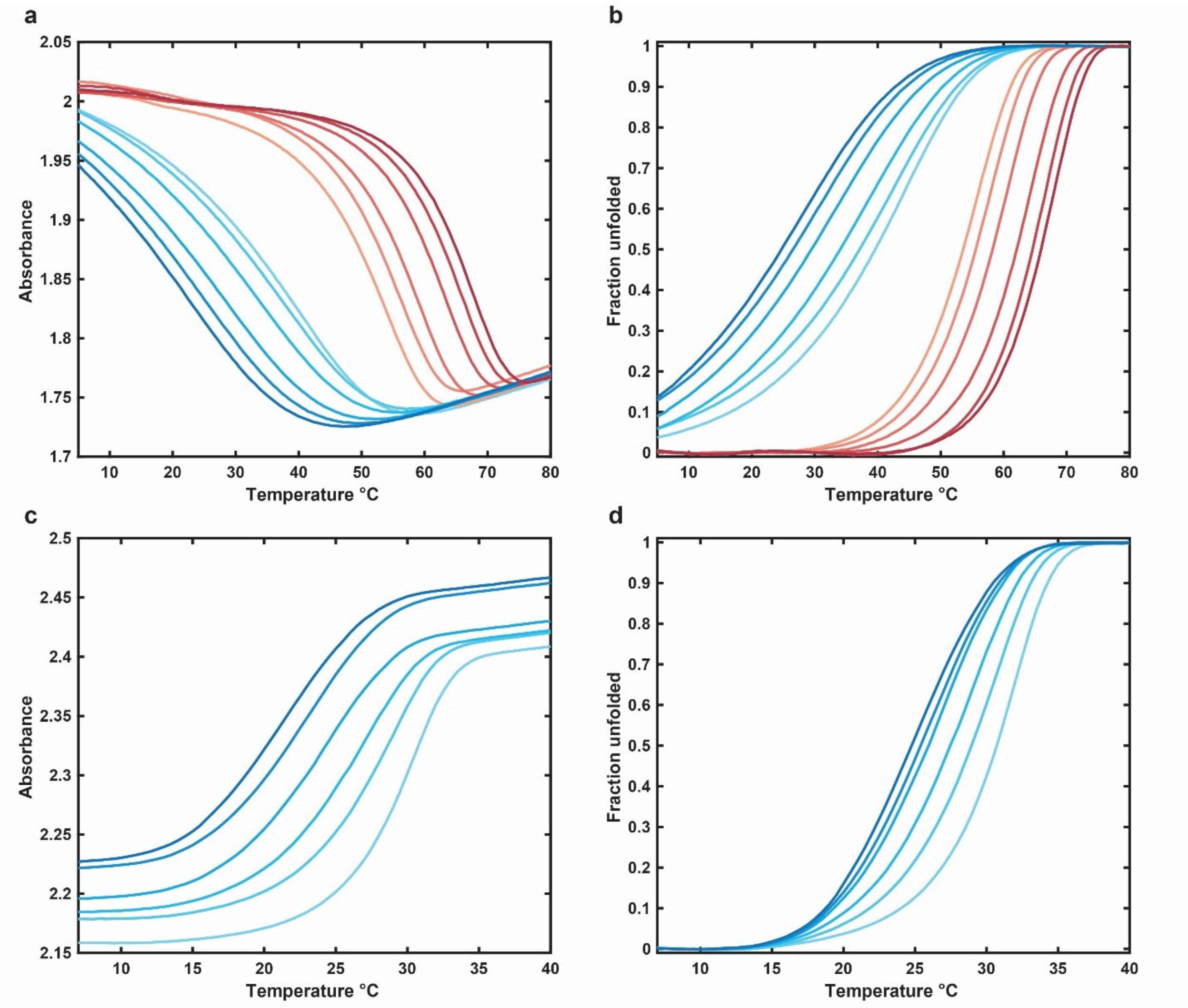

Supplementary Figure 3. Raw and corrected TH profiles. (a) Raw TG_4T absorbance data. (b) Baseline and temperature corrected TG_4T data used in all model-free and global fitting analyses. (c) Raw poly(A) fiber assembly TH profiles. (d) Baseline and temperature corrected poly(A) fiber assembly data used in all model-free and global fitting analyses. In all panels, dark to light blue indicate indicates fastest to slowest annealing scan rates, while dark red to light orange in (a,b) indicates fastest to slowest melting scan rates.

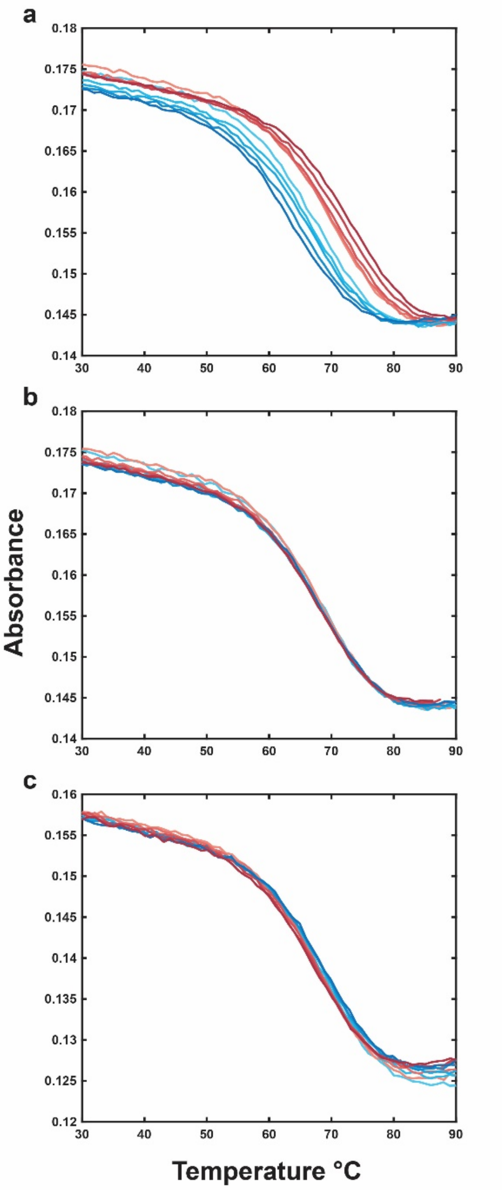

Supplementary Figure 4. Validation of the temperature correction of multi-scan rate thermal denaturation datasets. (a) Absorbance data collected by actively controlling the block temperature. (b) Data from (a) corrected as described in the Supplementary Methods. (c) Absorbance data collected by actively controlling the sample solution with an integrated temperature probe. Importantly, (b) and (c) produce nearly identical results, validating the temperature correction approach described here. The sample consisted of 5 μM of intramolecular G-quadruplex, 5’-AGGGTGGGIAGGGTGGGI-3’, in 10 mM lithium phosphate buffer pH 7.0 with 5 mM KCl. Experiments were measured with a Cary 300 UV-Visible spectrophotometer at 295 nm using scan rates of 1, 1.5, 2, 3, and 4 °C min^{-1}.

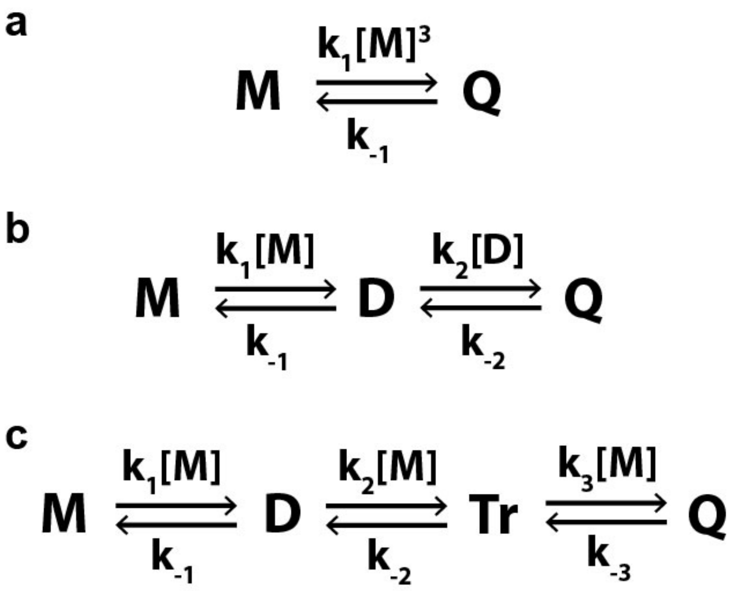

Supplementary Figure 5. Kinetic models of TG_4T assembly. (a) Concerted assembly of four monomer (M) strands to yield tetramer (Q). (b) The dimer-of-dimers (D) model. (c) Tetramer formation by step-wise monomer addition through dimeric and trimeric (Tr) intermediates.

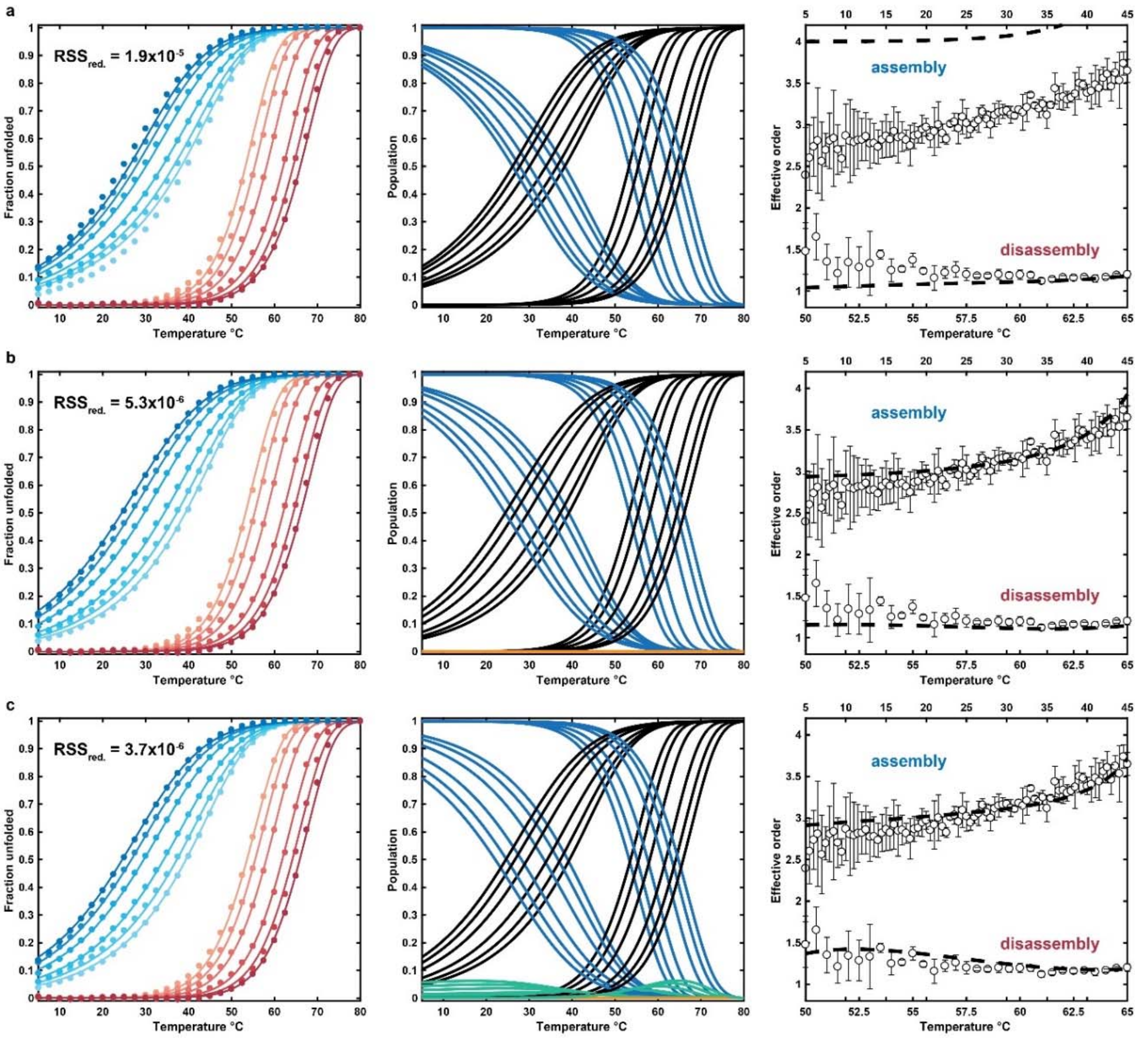

Supplementary Figure 6. Comparison of global fits of kinetic models to TG_4T TH profiles. (a) One-step assembly model (Supplementary Scheme 2a), $RSS_{red.} = 1.9\times10^{-5}$. (b) Dimer-of-dimers model (Supplementary Scheme 2b), $RSS_{red.} = 5.3\times10^{-6}$. (c) Step-wise monomer association model (Supplementary Scheme 1), $RSS_{red.} = 3.7\times10^{-6}$. For (a-c), panels show: (Left) Fraction unfolded TH profiles, where fits and experimental data are shown as colored lines and circles respectively. Only every 5th experimental point is shown for clarity. Dark to light blue corresponds to fastest and slowest annealing scan rates, and dark red to light orange corresponds to fastest to slowest melting scan rates respectively. The reduced *RSS*, $RSS_{red.}$ was calculated as RSS_0/DF where DF = # points - # fitted parameters. (Middle) Populations, where black and dark blue lines correspond to monomer and tetramer respectively. In (b) and (c), orange lines correspond to dimer population. In (c), green lines correspond to trimer population. (Right) Effective orders obtained from model-free analysis of experimental and fitted data, shown as white circles and dashed black lines respectively. The error bars for the experimental points are the standard deviation of model-free analysis on three replicate TH experiments.

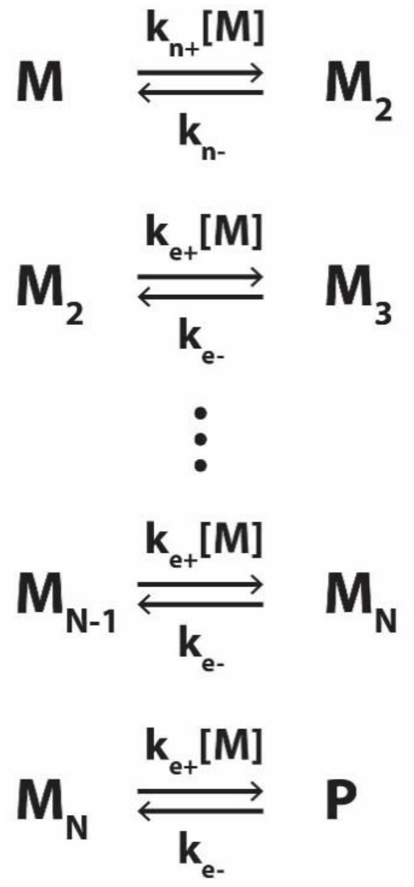

Supplementary Figure 7. The Goldstein-Stryer model for cooperative self assembly. The monomer (M) associates in a step-wise manner to form a nucleus of a defined size which then elongates to give large assemblies. The two regimes are defined by nucleation and elongation rate constants. The case for a nucleus size of 2 is shown here. We allowed post-nucleus oligomers to elongate up to an explicitly described size of N, beyond which they are treated as a fibril pool (P) according to the approximation by Korevaar *et al*[5, 6].

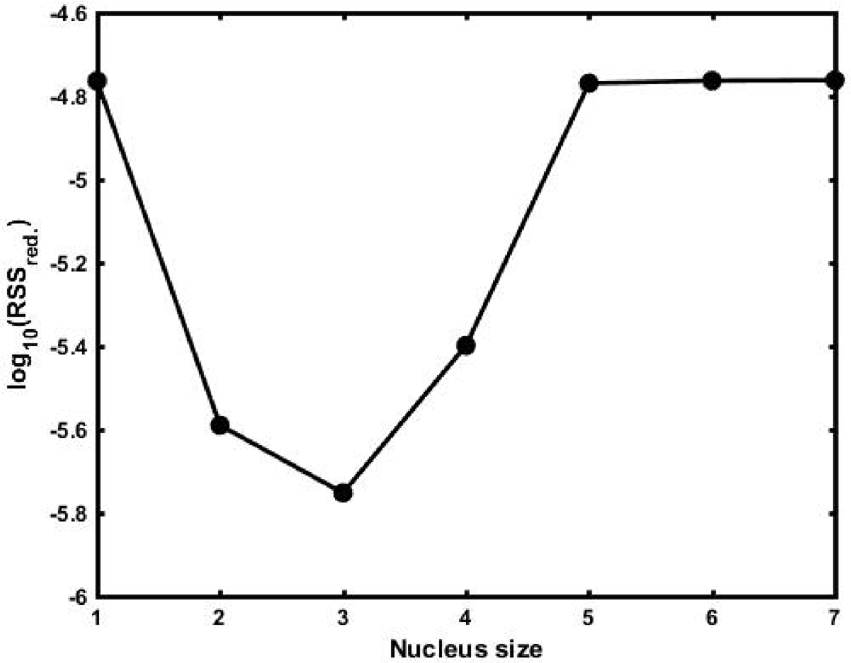

Supplementary Figure 8. Global fit quality as a function of nucleus size for global fits to CA-mediated poly(A) assembly TH profiles. The reduced *RSS*, $RSS_{red.}$, was calculated as RSS_0/DF, where *DF* = # points - # fit parameters. The nucleus size of 1 corresponds to a fit with an isodesmic (non-cooperative) mechanism where $k_{e+} = k_{n+}$ and $k_{e-} = k_{n-}$. The best fit was obtained with a nucleus size of 3.

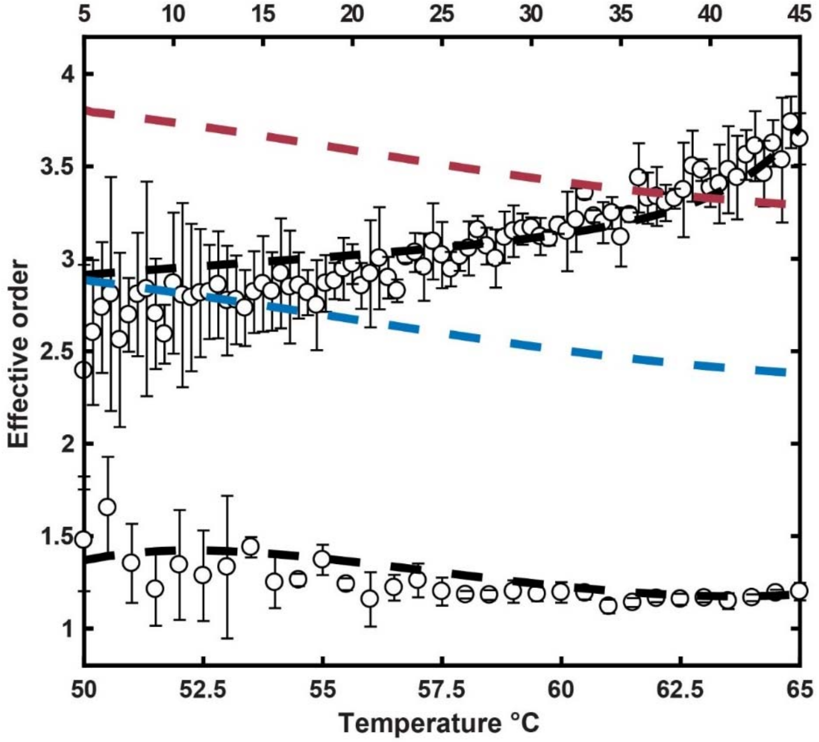

Supplementary Figure 9. Assessing the concentration dependence of the TG_4T assembly reaction orders at low and high temperature. The dark red and blue dashed lines are the assembly orders simulated with the step-wise model at 45 and 5 °C respectively as described in the Supplementary Methods section "Calculating apparent reaction orders".

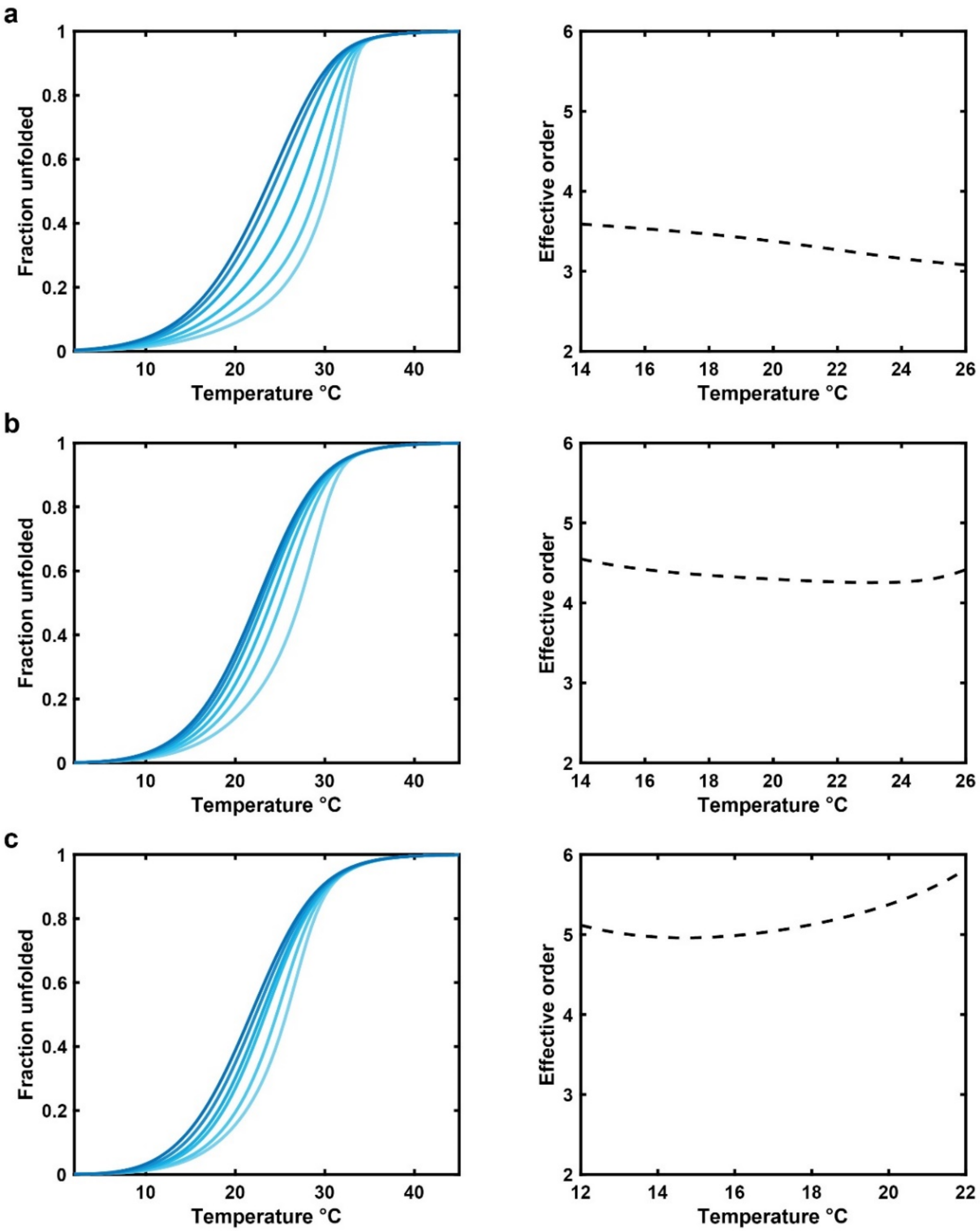

Supplementary Figure 10. Simulations of Goldstein-Stryer TH profiles as a function of nucleus size with fixed kinetic parameters. (a) Nucleus size of 3. (b) Nucleus size of 4. (c) Nucleus size of 5. In all left panels, dark to light blue indicates fastest to slowest annealing scan rates respectively. In all right panels, the effective assembly reaction order is shown as dashed black lines. Simulation parameters were E_{n+} = -27, k_{n+} = 1.75×10^4, E_{n-} = 35, k_{n-} = 2.4, E_{e+} = 50, k_{e+} = 7.5×10^4, E_{e-} = 90, k_{e-} = 0.5. Activation energies are given in kcal mol^{-1} and forward and reverse rate constants are given in M^{-1} min^{-1} and min^{-1} respectively at the reference temperature of 25 °C. Simulations were performed with $\Delta C_p^{\ddagger} = 0$ for all steps. The scan rates were (a,b) 0.2, 0.5, 1, 2, 3, 4 and (c) 0.1, 0.2, 1, 2, 4, 6 °C min^{-1}.

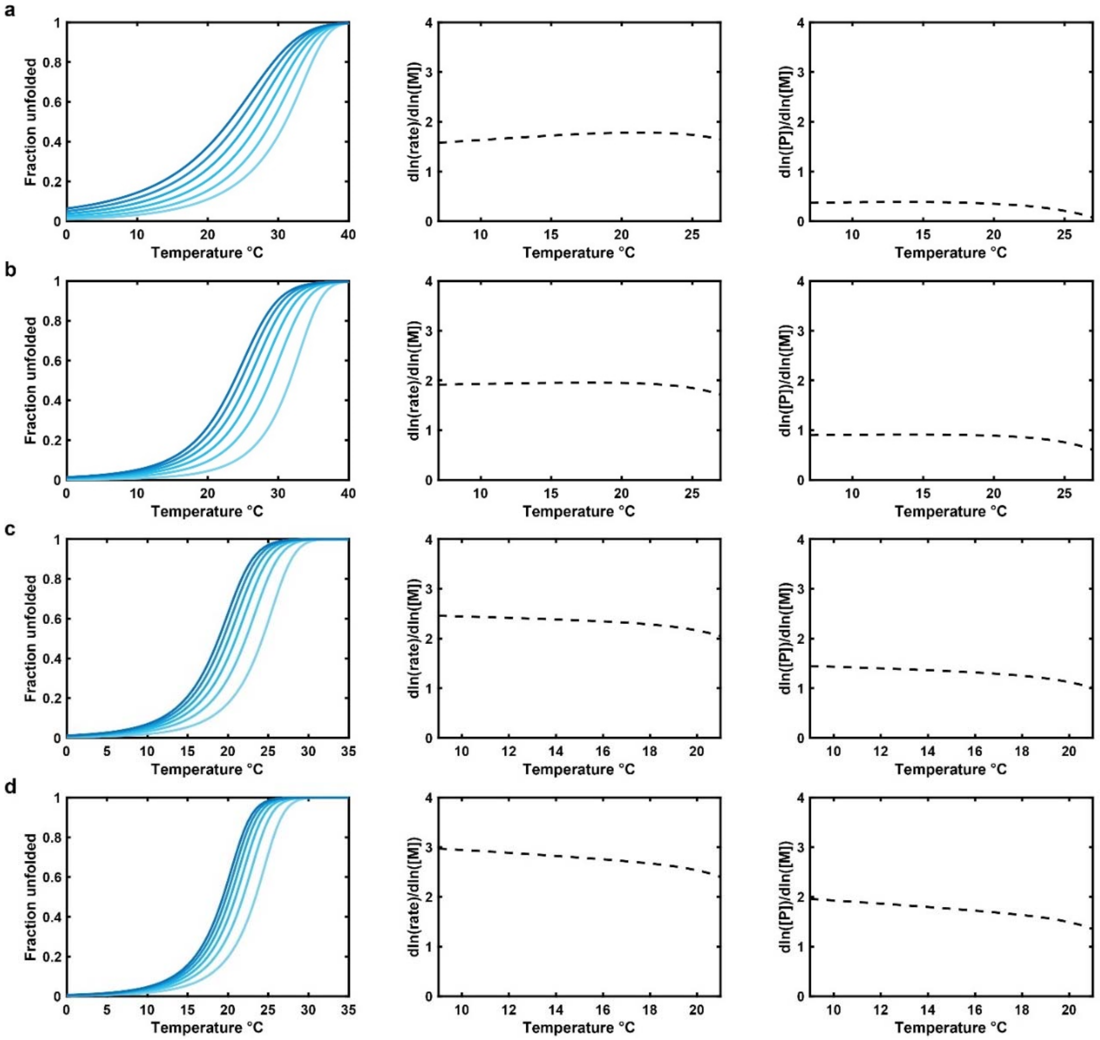

Supplementary Figure 11. Simulations of TH profiles for classical nucleated polymerizations. (a) Nucleus size of 2, ΔH_n = -30, K_n = 143, E_{e+} = 5, k_{e+} = 3.5×10^4, E_{e-} = 30, k_{e-} = 0.2. (b) Nucleus size of 3, ΔH_n = -40, K_n = 2×10^3, E_{e+} = 5, k_{e+} = 1×10^5, E_{e-} = 50, k_{e-} = 0.1. (c) Nucleus size of 4, ΔH_n = -40, K_n = 2×10^3, E_{e+} = 5, k_{e+} = 1×10^5, E_{e-} = 60, k_{e-} = 0.3. (d) Nucleus size of 5, ΔH_n = -40, K_n = 2×10^3, E_{e+} = 5, k_{e+} = 3×10^5, E_{e-} = 60, k_{e-} = 1. In all left panels, dark to light blue lines indicate fastest to slowest annealing scan rates respectively. Effective monomer reaction rate orders are shown in the middle panels as dashed black lines, tracking approximately as (s+1)/2 where s is the nucleus size. Fiber concentration ([P]) orders are shown in the right panels as dashed black lines, tracking approximately as (s-1)/2. Nucleation ΔHs and elongation activation energies are given in kcal mol^{-1}, nucleation equilibrium constants are given in M^{-1}, and forward and reverse rate constants are given in M^{-1} min^{-1} and min^{-1} respectively at the reference temperature of 25 °C. The critical monomer concentration $[M]_{critical}$ was calculated as k_{e-}/k_{e+}. Simulations were performed with $\Delta C_p^{\ddagger}$ = 0 for all steps. In (a), scan rates were 0.2, 0.3, 0.4, 0.5, 0.6, and 0.7 °C min^{-1}. In (b-d), scan rates were 0.2, 0.4, 0.6, 0.8, 1, and 1.2 °C min^{-1}.

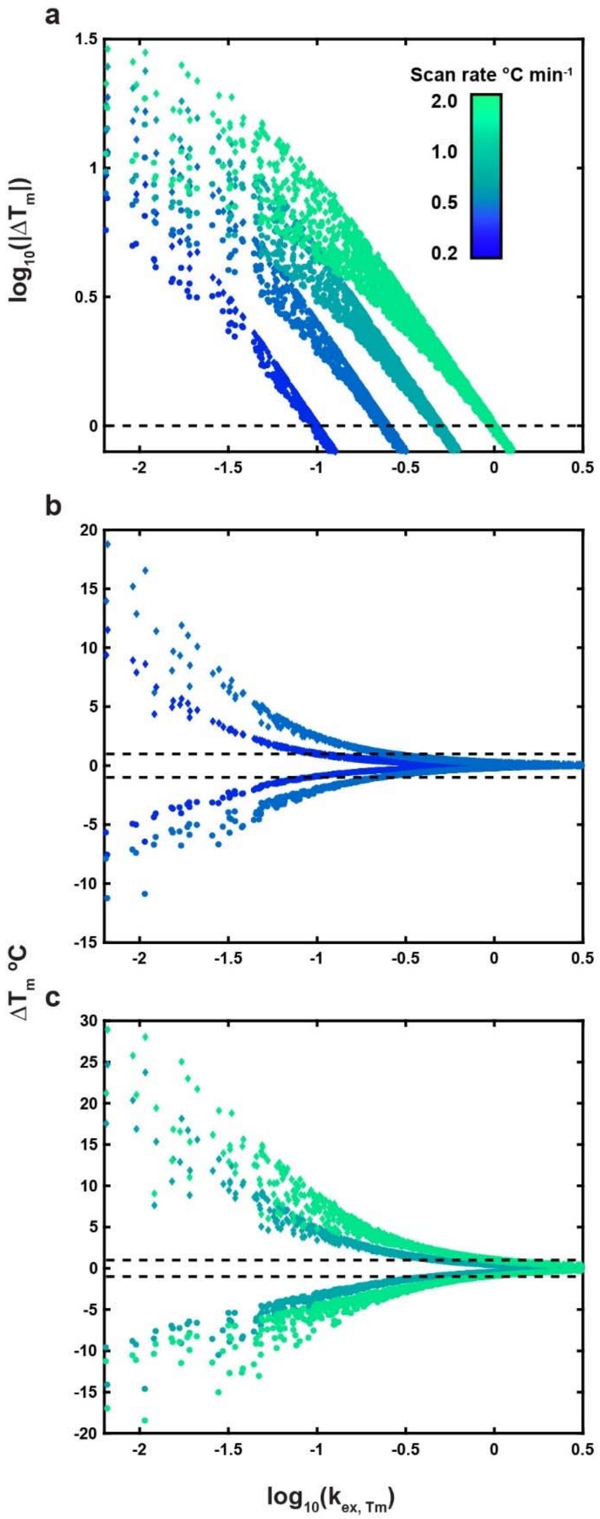

Supplementary Figure 12. Assessing the extent of TH as a function of assembly and disassembly kinetics for a dimeric system $2M \leftrightarrow M_2$. **(a)** $Log_{10}(|\Delta T_m| = |T_{50\%} - T_m|)$ (where $T_{50\%}$ and T_m are the

50% assembled or disassembled and equilibrium melting temperatures respectively) versus $\log_{10}(k_{ex}$ (min^{-1}) $= k_f[M] + k_u)$ at the T_m (i.e. $[M] = 2[M_2]$, $k_fC_T = k_u$, $k_{ex}=(1/2)C_Tk_f + C_Tk_f = (3/2)C_Tk_f)$ for 0.2, 0.5, 1, and 2 °C min^{-1} scan rates. (b,c) $\Delta T_m = T_{50\%} - T_m$ versus $\log_{10}(k_{ex})$ at the T_m for **(b)** 0.2, 0.5, **(c)** 1, and 2 °C min^{-1} scan rates. 1000 noiseless TH datasets were randomly generated using a total monomer concentration C_T = 10 µM and simulation parameters drawn from uniform probability distributions over the intervals [-10, -80] kcal mol^{-1}, [50, 5×10^5] M^{-1} min^{-1}, [20, 90] kcal mol^{-1}, and [5×10^{-2}, 5×10^{-5}] min^{-1} for E_f, $k_{f,0}$, E_u, and $k_{u,0}$ respectively. T_m values were calculated according to $T_m = \Delta H/[\Delta S + R\ln(C_T)]$. ΔH and ΔS of association were calculated as $(E_f - E_u)$ and $(\Delta H/T_{ref} + R\ln(k_{f,0}/k_{u,0}))$ respectively. Temperature dependent k_f and k_u values were calculated according to Eq. 13 (above) and T_{ref} was set to 298 K. Simulated annealing and melting data are plotted as filled circles and diamonds respectively. Dark blue to light green symbols indicate slowest to fastest scan rates respectively. Dashed black lines indicate $\Delta T_m = \pm 1$ °C, an approximate lower limit for reliable analysis. Note that kinetics are highly tunable by varying C_T, since $k_{ex}=(3/2)k_fC_T$ at the mid-point of the transition. Reducing the total monomer concentration by a factor of 10 decreases the assembly kinetics by a factor of 10 in the transition region relevant to TH experiments.

Supplementary Tables

Supplementary Table 1. TH global fit parameters for TG_4T assembly with the step-wise monomer association model. Activation energies are given in kcal mol^{-1}. Rate constants are given at the reference temperature of 45 °C and in M^{-1} min^{-1} and min^{-1} for forward and reverse steps respectively. Errors were calculated according to the variance-covariance method[10] as stated in the Supplementary Methods.

Activation energies		Rate constants	
E_1	-5.4 ± 0.8	k_1	(3.0 ± 0.3) × 10^2
E_{-1}	14.4 ± 0.4	k_{-1}	(5.0 ± 1.0) × 10^3
E_2	-4.0 ± 1.2	k_2	(1.6 ± 0.2) × 10^5
E_{-2}	15. 9 ± 0.4	k_{-2}	(3.1 ± 0.2) × 10^{-1}
E_3	-3.8 ± 0.7	k_3	(8.2 ± 0.5) × 10^2
E_{-3}	37.4 ± 0.2	k_{-3}	(8.4 ± 0.1) × 10^{-3}

Supplementary Table 2. TH global fit parameters for poly(A) fiber assembly using the Goldstein-Stryer model[4] with a nucleus size of 3. Activation energies are given at the reference temperature of 25 °C in kcal mol^{-1}. Activation heat capacities are given in kcal mol^{-1} K^{-1}. Rate constants are given at the reference temperature of 25 °C and in M^{-1} min^{-1} and min^{-1} for forward and reverse steps respectively. Errors were calculated according to the variance-covariance method[10] as stated in the Supplementary Methods.

Activation energies		Rate constants		Heat capacities	
$E_{n+} = E_{e+}$	14.5 ± 1.1	$k_{n+} = k_{e+}$	(6.7 ± 0.2) × 10^4	$\Delta C_p{}^{‡}{}_{n+} = \Delta C_p{}^{‡}{}_{e+}$	5.2 ± 0.3
E_{n-}	96.4 ± 2.0	k_{n-}	6.8 ± 0.3	$\Delta C_p{}^{‡}{}_{n-}$	5.5 ± 0.5
E_{e-}	133.0 ± 0.8	k_{e-}	(5.8 ± 0.4) × 10^{-2}	$\Delta C_p{}^{‡}{}_{e-}$	0.5 ± 0.2

Supplementary References

1. Bardin, C. & Leroy, J.L. The formation pathway of tetramolecular G-quadruplexes. *Nucleic Acids Res.* **36**, 477-488 (2008).

2. Mergny, J.L., De Cian, A., Ghelab, A., Sacca, B. & Lacroix, L. Kinetics of tetramolecular quadruplexes. *Nucleic Acids Res.* **33**, 81-94 (2005).

3. Wyatt, J.R., Davis, P.W. & Freier, S.M. Kinetics of G-quartet-mediated tetramer formation. *Biochemistry* **35**, 8002-8008 (1996).

4. Goldstein, R.F. & Stryer, L. Cooperative polymerization reactions. Analytical approximations, numerical examples, and experimental strategy. *Biophys. J.* **50**, 583-599 (1986).

5. Korevaar, P.A. et al. Pathway complexity in supramolecular polymerization. *Nature* **481**, 492-496 (2012).

6. van der Zwaag, D. et al. Kinetic analysis as a tool to distinguish pathway complexity in molecular assembly: an unexpected outcome of structures in competition. *J. Am. Chem. Soc.* **137**, 12677-12688 (2015).

7. Mikulecky, P.J. & Feig, A.L. Heat capacity changes associated with DNA duplex formation: salt- and sequence-dependent effects. *Biochemistry* **45**, 604-616 (2006).

8. Tikhomirova, A., Taulier, N. & Chalikian, T.V. Energetics of nucleic acid stability: the effect of DeltaCP. *J. Am. Chem. Soc.* **126**, 16387-16394 (2004).

9. Fersht, A. Structure and mechanism in protein science: a guide to enzyme catalysis and protein folding. (W.H. Freeman, New York; 1999).

10. Tellinghuisen, J. Statistical error propagation. *J. Phys. Chem. A* **105**, 3917-3921 (2001).
